# Decoding steroid-derived metabolite engineering in *Solanum*

**DOI:** 10.1093/hr/uhaf178

**Published:** 2025-07-07

**Authors:** Farah Kanwal, Sana Ullah, Yingchen Hao, Shouchuang Wang

**Affiliations:** National Key Laboratory for Tropical Crop Breeding, School of Breeding and Multiplication (Sanya Institute of Breeding and Multiplication), Hainan University, Sanya 572025, China; National Key Laboratory for Tropical Crop Breeding, School of Breeding and Multiplication (Sanya Institute of Breeding and Multiplication), Hainan University, Sanya 572025, China; National Key Laboratory for Tropical Crop Breeding, School of Breeding and Multiplication (Sanya Institute of Breeding and Multiplication), Hainan University, Sanya 572025, China; National Key Laboratory for Tropical Crop Breeding, School of Breeding and Multiplication (Sanya Institute of Breeding and Multiplication), Hainan University, Sanya 572025, China; National Key Laboratory for Tropical Crop Breeding, College of Tropical Agriculture and Forestry, Hainan University, Sanya 572025, China; Yazhouwan National Laboratory, Sanya 572025, China

## Abstract

Steroidal glycoalkaloids (SGAs) and steroidal saponins (STSs) play significant role in the plant defence against pests and offer various pharmaceuticals applications. SGAs and STSs generally share common biosynthetic pathways in *Solanum*, originating from a furostanol scaffold. Despite the discovery of multiple GLYCOALKALOID METABOLISM (*GAME*) genes involved in the biosynthesis of these compounds, previous attempts for the metabolic engineering of these pathways have remained unsuccessful. The GAME15 protein, with its dual enzymatic roles, has unlocked a mystery surrounding the intricate process of metabolizing cholesterol. This protein not only acts as a glucuronosyltransferase but also serves as a metabolic scaffold, organizing several proteins for the proper functioning. This mini review briefly describes the molecular mechanisms and functional dynamics of *GAME* genes, particularly focusing on *GAME15* as a key game changer gene and its role in metabolite channelling, regulation of pathway, and ecological importance. We highlighted the potential of this discovery for advancing metabolic engineering in crop improvement and the pharmaceutical industry. This finding opens doors for designing crops that are resistant to pests. Additionally, we identify important future research directions, including the regulatory mechanisms of these pathways and uncovering structural aspects of pivotal enzymes.

## Introduction

Plants and fungi produce numerous secondary metabolites that play key roles in their development and regulate interactions with both allies and adversaries, tailored to their ecological functions [1–3]. Through specialized metabolism, the *Solanum*, which includes commercially significant crops like tomato, potato and eggplant, has developed complex chemical defence systems [4, 5]. Among these, steroidal glycoalkaloids (SGAs) and steroidal saponins (STSs), two major classes of cholesterol-derived metabolites, serve as essential defensive agents against pests and pathogens due to their inherent toxicity [6]. While SGAs are notably studied for their antinutritional effects in food crops, both classes also exhibit broader bioactive potential in therapeutic contexts. Phylogenetic studies further indicate distinct evolutionary divergence in SGA/STS biosynthesis pathways within the *Solanum*, reflecting adaptation to ecological pressures [7, 8].

**Figure 1:**
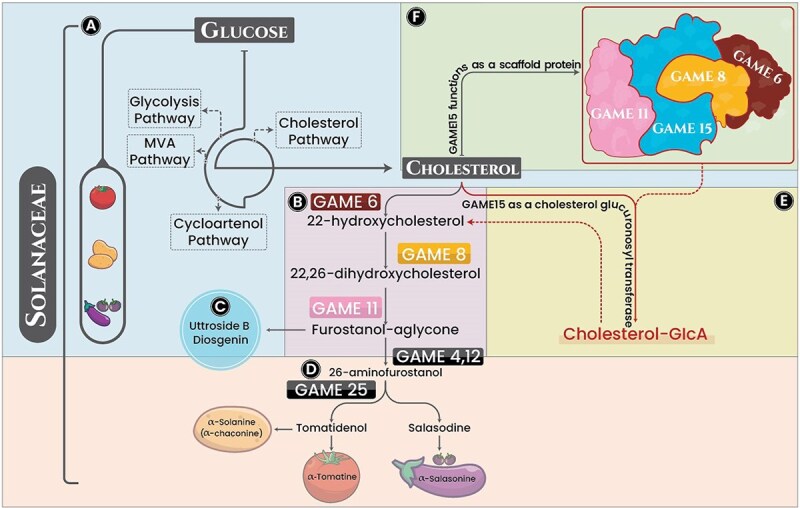
Schematic diagram to quickly understand SGA biosynthesis pathway before and after discovery of GAME15. A) Pathway of cholesterol formation from glucose through glycolysis, cytosolic isoprenoid mevalonate pathway and the cycloartenol pathway. B) Common pathway of cholesterol transformation initiation to steroidal alkaloid formation through multiple hydroxylation by GAME6, GAME8 and GAME11 to from furostanol-aglycone scaffold enzyme. C) Furostanol-aglycone converts into STS or D) Specie-specific conversion of Furostanol-aglycone to SGAs through GAME4, GAME11 and GAME25. E) Role of GAME15 as scaffold protein to form complex with GAME6, GAME8, and GAME11. F) GAME15 function as a glucuronosyltransferase by adding glucuronic acid to cholesterol.

Although SGAs are primarily recognized for their antinutritional properties in food crops, they exhibit a remarkable spectrum of bioactive potential, including anticancer, antimicrobial, anti-inflammatory, antiviral and antipyretic activities [8, 9]. Their defensive function is epitomized by dominant SGAs in key crops: α-tomatine in tomato, α-chaconine in potato and α-solanine in eggplant, all acting as natural pesticides against herbivores and pathogens [10]. Similarly, STSs have emerged as compounds of substantial industrial significance. Their detergent-like properties have made them valuable targets for pharmaceutical, cosmetic and food industries [11]. Notably, STSs, such as uttroside B, share a common furostanol scaffold with SGAs, suggesting that early biosynthetic steps are shared between these pathways.

The biosynthetic pathways of these specialized metabolites have been extensively studied, leading to significant progress in identifying and characterizing key GLYCOALKALOID METABOLISM (*GAME*) genes involved in SGA and STS biosynthesis (Fig. 1) [7, 12, 13]. These *GAME* genes were largely identified via different approaches including comparative co expression analysis, which was linked to the SGA accumulation metabolomics, and chemical profiling ([7]. Early enzymatic steps-including hydroxylations catalyzed by GAME6, GAME8 and GAME11—generate the furostanol-type aglycone scaffold, a central intermediate for both compound classes [14, 15]. This process occurs through dynamic enzyme–enzyme assemblies that enhance efficiency by channelling toxic intermediates. The metabolons are defined as the enzyme–enzyme complexes, which are involved in enhancing the metabolic efficiency while preventing the accumulation of toxic intermediate compounds and maintaining the substrate concentration. These complexes facilitate local substrate concentration, prevent side reactions, and protect cells from toxic intermediates [16–18]. Steroid biosynthesis is the only specialized metabolic process in which GAME15 supports the temporary groups working with intermediates. Studies conducted recently point to GAME15 organizing a PPI network alongside GAME6, GAME8 and GAME11, which improves metabolic activity [19]. Despite decades of research, the incomplete knowledge of early pathway steps persistently hindered heterologous reconstitution of SGA biosynthesis [7, 15]. The discovery of GAME15 resolved this impasse as the long-missing enzyme essential for initiating cholesterol conversion [10, 20]. As a dual-function cellulose synthase-like (CSL) protein localized to the ER, it catalyzes initial glucuronidation while organizing metabolic complexes [19]. Beyond filling a critical knowledge gap in *Solanum* metabolism, this breakthrough provided the evidence for STS roles in plant defence and enabled the first successful heterologous reconstitution of the full SGA aglycone pathway [19, 21, 22], opening new avenues for metabolic engineering in crop protection and pharmaceutical development.

In this review, we summarized the current understanding of *GAME* members identified in *Solanum*, emphasizing the critical role of GAME15 in steroidal metabolite biosynthesis. We explore its dual enzymatic and structural functions, dissect the molecular mechanisms of metabolon formation, and discuss the ecological and agricultural significance of SGAs and STSs. Finally, we highlight emerging applications in crop improvement and drug development enabled by the ground-breaking discovery of GAME15.

## The molecular architect of steroidal metabolite biosynthesis

In *Solanum* plants, the biosynthesis of SGA and STS originates from cholesterol as the central precursor. This biosynthetic mechanism initiates from glucose by proceeding through sequential pathways including glycolysis, the cytosolic isoprenoid mevalonate pathway, and cycloartenol pathway [23]. The final step in cholesterol formation is catalyzation of cycloartenol by sterol side chain reductase 2 (SSR2) through its D24reductase activity, which converts cycloartenol to cholesterol, thereby initiating the complex biochemical transformations required for steroidal alkaloid biosynthesis[16, 24]. The molecular mechanism of cholesterol transformation revealed a series of hydroxylation reactions catalyzed by different GAME family members, which comprises of several members with diverse functions in the SGA biosynthetic pathway, as summarized in Table 1. For instance, the cytochrome P450 enzyme GAME6 that performs C22 hydroxylation, the cytochrome P450 enzyme GAME8 catalyzing C26 hydroxylation, and the dioxygenase GAME11 that conducts C16 hydroxylation resulting in the formation of a furostanol-type aglycone scaffold [25,26]. This furostanol scaffold serves as a critical branching point in the pathway, directing metabolites toward either STSs or SGAs. This scaffold either forms STSs such as uttroside B or undergoes some chemical modifications of oxidization to 26-furostanol aldehyde by cytochrome P450 enzyme GAME4, which further undergoes the putative transaminase GAME12-mediated conversion to 26-aminofurostanol. The aminofurostanol intermediate is then cyclized by GAME25, initiating species-specific SGA biosynthesis [10]. The short-chain dehydrogenase/reductase GAME25 helps in the production of specific compounds in certain plants. It changes 26-aminofurostanol into substances like α-tomatine in tomatoes, α-solanine and α-chaconine in potatoes, and α-solasonine and α-solamargine in eggplants and black nightshade [10,27]. Notably, the SGA profiles are dynamically regulated during fruit maturation. Ripening-associated gene clusters (e.g. *GAME31, GAME36, GAME40*) convert toxic α-tomatine into less harmful derivatives like esculeoside A in tomatoes [28,29]. Recent work has further characterized the complete set of enzymes involved in subsequent decorations of steroidal scaffolds in *Solanum* species, including the UGTs leading to α-solasonine and related compounds [8].

**Table 1:** *GAME* genes identified in *Solanum* plants and their functions in SGAs and STSs biosynthesis pathways.

**Gene names**	**Species**	**Functions**	**Pathway**	**Reference**
*GAME1*	*S. lycopersicum*	UDP-galactosyltransferase; converts tomatidine (steroidal alkaloid aglycone) to tomatidine-galactoside (T-Gal), initiating glycosylation in α-tomatine synthesis	SGAs	[30]
*GAME2*	*S. lycopersicum*	UDP-xylosyltransferase that catalyzes the addition of xylose to β1-tomatine during SGA biosynthesis	SGAs only	[31]
*GAME4/Cytochrome P450* *CYP88B1*	*S. tuberosum*, *S. lycopersicum*	Cytochrome P450; oxidizes furostanol-type intermediate to its 26-aldehyde, enabling nitrogen incorporation via transamination (by GAME12) into SGAs	SGAs and STSs	[7]
*GAME6/CYP72A188*	*S. lycopersicum*, *S. tuberosum*	Cytochrome P450; proposed to contribute to furostanol aglycone formation, likely oxidizing at C22 and aiding E-ring closure with GAME11	SGAs	[7]
*GAME8/CYP72A208*	*S. lycopersicum*, *S. tuberosum*	Cytochrome P450; hydroxylates cholesterol at C26, forming 22,26-dihydroxycholesterol after GAME7 action	SGAs	[7]
*GAME9/JRE4*	*S. lycopersicum*, *Solanum tuberosum*	AP2/ERF transcription factor; regulates the biosynthesis of SGAs and upstream cholesterol biosynthesis by controlling expression of SGA biosynthetic genes (e.g. GAME1, GAME4) and cholesterol pathway genes (e.g. C5-SD, SSR2)	SGAs, Cholesterol, Mevalonate	[22]
*GAME11*	*S. lycopersicum*	Putative dioxygenase; hydroxylates 22,26-dihydroxycholesterol at C16 and, with GAME6, facilitates E-ring closure to form furostanol-type aglycone	SGAs	[7]
*GAME12*	*S. lycopersicum*, *S. tuberosum*	Transaminase: transaminates 26-aldehyde (from GAME4) to tomatoxine, which is dehydrogenated to tomatidine	SGAs	([6]; [7])
*GAME15*	*S. nigrum*, *S. lycopersicum*, *S. tuberosum*, *S. melongena*	Scaffold protein; interacts with early enzymes (GAME6, GAME8, GAME11) to enable furostanol production	Both (Saponins and SGAs)	[10]
*GAME17*	*S. lycopersicum*	UDP-glucosyltransferase; adds glucose to T-Gal, forming γ-tomatine	SGAs	[7]
*GAME18*	*S. lycopersicum*	UDP-glucosyltransferase; adds glucose to γ-tomatine, forming β1-tomatine	SGAs	[7]
*GAME25*	*S. lycopersicum*, *S. dulcamara*, *S. chacoense*, *S. demissum*, *S. commersonii*, *S. melongena*	Short-chain dehydrogenase/reductase; catalyzes the first step in a proposed three-step reduction of the C-5,6 double bond, converting dehydrotomatidine to tomatidine (tomato), solanidine to demissidine (wild potatoes), and solasodine to soladulcidine (wild *Solanum* spp.); exhibits 3β-hydroxysteroid dehydrogenase/Δ^5^,^4^ isomerase activity on steroidal alkaloid and saponin aglycones	SGAs and STSs	[27]
*GAME31*		2-Oxoglutarate-dependent dioxygenase; catalyzes the hydroxylation of the bitter α-tomatine to hydroxytomatine	SGAs	[32]
*GAME34*	*S. habrochaites*, *S. pennellii*, *S. lycopersicum*	2-Oxoglutarate-dependent dioxygenase; converts α-tomatine to habrochaitoside A and dehydrotomatine to habrochaitoside B; also acts on tomatidine to form habrochaitoside C	SGAs only	[33]
*GAME35*	*S. habrochaites*, *S. lycopersicum*	2-Oxoglutarate-dependent dioxygenase; no detectable activity with tested SGA/SA substrates (e.g. α-tomatine, dehydrotomatine, tomatidine, α-solanine, α-chaconine, solanidine)	NA	[33]

Recent breakthrough studies by Jozwiak et al. [19] and Boccia et al. [10] have identified GAME15 as a crucial bifunctional enzyme in SGA and STS biosynthesis. This endoplasmic reticulum-localized cellulose synthase-like protein acts as both a glucuronosyltransferase attaching glucuronic acid to cholesterol, and a scaffold protein enabling pathway reconstitution.

## GAME15 discovery: a way to biosynthetic engineering

Despite the identification and characterization of these GAME enzymes in tomato and potato, attempts to reconstitute SGA biosynthesis in heterologous hosts like *Nicotiana benthamiana* have been unsuccessful [20]. This failure suggests that the known GAME enzymes (GAME6, GAME8, GAME11, GAME4 and GAME12) represent an incomplete picture of the biosynthetic pathway, indicating additional undiscovered components are likely essential for steroidal metabolite production in *Solanum* species.

The bifunctionality of GAME15 was established through in vitro microsomal assays and in vivo studies, revealing GAME15 as an endoplasmic reticulum (ER)-localized glucuronosyltransferase with unique substrate specificity—broad activity in yeast (e.g. ergosterol, lanosterol) but exclusive cholesterol glucuronidation in planta. Protein interaction analyses (split-luciferase and FLIM-FRET) confirmed GAME15 scaffolds a metabolon with early-pathway enzymes (GAME6/GAME8) and cholesterol biosynthesis proteins (7-DR2/C5-SD2), enhancing intermediate channelling while restricting diffusion of cytotoxic compounds [19,34]. Critically, deuterium-labelling experiments in *N. benthamiana* showed dramatically reduced incorporation of external cholesterol when GAME15 was present, directly demonstrating metabolon-mediated substrate channelling and explaining prior reconstitution failures [19,35].

Functional validation through knockout *GAME15*-knockout experiments in tomato and potato demonstrated GAME15’s essential role. The *GAME15* mutants exhibited complete ablation of both SGAs and STSs, with concomitant cholesterol accumulation [10]. These *GAME15*-knockout plants showed increased susceptibility to insect pests, particularly against *Empoasca decipiens* and Colorado potato beetle, providing the first definitive evidence for STS role in plant defence [10,25]. Choice and feeding experiments with different pests confirmed this ecological function, wild-type leaves remained unpalatable, while leaves depleted of STSs were highly susceptible to flavoury, confirming the role of STSs in plant defence.

Similar knockout experiments in *S. nigrum* confirmed these findings, the critical role of GAME15 in both metabolite biosynthesis and plant defence against herbivores was further validated, reinforcing its indispensable function in these biological processes. With GAME15 identified as the key missing component, researchers successfully reconstituted the complete SGA and STS biosynthetic pathways. Jozwiak et al. [19] demonstrated that co-expression of GAME15with the catalytic enzymes was essential for pathway reconstitution in *N. benthamiana*. This essential function was further confirmed by the transient reconstitution of the SGA biosynthetic pathway in *N. benthamiana*, showing that GAME15 restored the bioproduction of SGAs in a heterologous system, which was not possible with the sole overexpression of *GAME6/8/11* genes. In the absence of GAME15, even when all other enzymes were present, the metabolic flux was disrupted, and target metabolites were not produced. This finding resolved the long-standing puzzle of why previous metabolic engineering efforts failed despite the expressing all known catalytic enzymes. GAME15 acts as a scaffold protein, providing a large entropic benefit by increasing the effective concentration of enzymes and substrates [19]. This results in the formation of an efficient metabolon, a supramolecular assembly that coordinates the complex, multistep reactions of the biosynthetic pathway.

This breakthrough opens numerous possibilities for metabolic engineering applications, with implications spanning pharmaceutical production, crop improvement and synthetic biology. The successful reconstitution of complete SGA/STS pathways in heterologous hosts, such as *N. benthamiana*, enables scalable production of pharmaceutically valuable steroidal compounds. A prime example is uttroside B, an FDA-approved orphan drug for hepatocellular carcinoma treatment [36], whose reliable supply can now be secured through engineered biological systems. The metabolic engineering of crops to optimize SGA/STS profiles presents a dual benefit: enhancing natural pest resistance while mitigating antinutritional steroidal alkaloids in edible tissues. This approach aligns with sustainable agriculture goals by reducing pesticide reliance without compromising crop edibility. In addition, understanding the subcellular organization of GAME enzymes provides the way for the bioproduction of steroidal metabolites in tailored ‘cell factories’ for securing a supply of the anticancer agent uttroside B. Looking forward, synthetic biology strategies could leverage GAME15’s dual functional roles in enzyme scaffolding and metabolic flux coordination to design novel metabolons. Such engineered supramolecular assemblies could enable the biosynthesis of structurally modified or entirely novel steroidal compounds, optimized for enhanced bioactivity in medicinal contexts or improved agronomic traits in crop breeding [37].

## Unanswered questions

- Molecular mechanisms of bifunctionality: How does game15 coordinate its dual functions as both enzyme and scaffold? What structural features enable this bifunctionality?
- Evolutionary origin of cellulose synthase homologs: What selective pressures drove the repurposing of a cellulose synthase-like protein into a core component of specialized metabolism? Are there intermediate evolutionary forms in related species?
- Transcriptional and spatiotemporal regulation: Which transcription factors coordinate SGA biosynthesis with developmental cues and environmental stresses? How is tissue-specific expression regulated?
- Metabolic compartmentalization and toxicity control: What glycosidase removes the GlcA moiety during biosynthesis, and where is this reaction localized? How does subcellular compartmentalization prevent toxicity from pathway intermediates?
- Engineering optimization: What are the optimal expression levels and subcellular localization patterns for GAME15 and associated enzymes in heterologous hosts? How can metabolic flux be balanced with potential toxicity?

## Conclusion and future perspectives

The identification of GAME15 as a scaffold-catalytic hub marks a transformative milestone in plant specialized metabolism research, resolving the long-standing challenge of SGA pathway reconstitution in heterologous hosts. To address the molecular mechanisms of GAME15’s dual functionality, structural biology approaches including crystallography and cryo-electron microscopy could reveal specific domains enabling both enzymatic catalysis and metabolon assembly, including transmembrane regions for membrane anchoring or protein-interaction interfaces for multienzyme complex formation. Comparative genomics across *Solanum* may illuminate the evolutionary journey of CSL homologs from cell wall biosynthetic enzyme to specialized metabolism, uncovering intermediate forms with partial scaffold or catalytic activities that provide insights into how primary metabolism enzymes are recruited for specialized functions. Such delocalization likely led to functional convergence with other enzymes involved in specialized metabolism, resulting in the evolution from their primary functions.

Systems biology approaches integrating transcriptomics, proteomics and metabolomics could elucidate the complex regulatory networks controlling SGA biosynthesis in response to developmental cues and environmental stresses. Identifying the glycosidase that removes the GlcA moiety and understanding its subcellular localization would provide valuable insights into metabolic control mechanisms. For engineering applications, determining optimal expression levels and stoichiometric ratios of GAME15 and associated enzymes, while developing strategies to minimize flux bottlenecks without accumulating toxic intermediates, will be essential for commercial viability. This organizational principle resembles other plant metabolons such as those in isoflavonoid biosynthesis [38], suggesting a common strategy for pathway optimization.

The successful reconstitution of SGAs and STSs pathways in *N. benthamiana* not only validates GAME15 as a core metabolon component but also establishes a scalable synthetic biology platform. The growing importance of GAME15 in both fundamental and applied research underscores how discoveries at the intersection of primary and specialized metabolism can advance both scientific understanding and biotechnological innovation. Future work examining the broader applicability of metabolon engineering in other specialized metabolic pathways may reveal whether the GAME15 paradigm represents a common evolutionary strategy or a unique solution to the challenges of complex secondary metabolism. The self-protective mechanisms that plants have evolved to manage these potent metabolites may also inspire novel strategies for targeted drug delivery systems in pharmaceutical applications, while exploring potential applications of engineered SGAs and STSs including biopesticides, functional foods and industrial surfactants. This research trajectory deepens our understanding of the evolution of plant chemical diversity while providing scientifically rigorous and practically viable solutions for sustainable biomanufacturing.
